# Postoperative Radioiodine Treatment within 9 Months from Diagnosis Significantly Reduces the Risk of Relapse in Low-Risk Differentiated Thyroid Carcinoma

**DOI:** 10.1007/s13139-019-00608-8

**Published:** 2019-09-05

**Authors:** Jolanta Krajewska, Michal Jarzab, Aleksandra Kukulska, Agnieszka Czarniecka, Jozef Roskosz, Zbigniew Puch, Zbigniew Wygoda, Ewa Paliczka-Cieslik, Aleksandra Kropinska, Aleksandra Krol, Daria Handkiewicz-Junak, Barbara Jarzab

**Affiliations:** 1 Nuclear Medicine and Endocrine Oncology Department, M.Sklodowska-Curie Institute–Oncology Center, Gliwice Branch, Wybrzeze AK 15, 44-101 Gliwice, Poland; 2 IIIrd Radiotherapy Clinic, M.Sklodowska-Curie Institute–Oncology Center, Gliwice Branch, Gliwice, Poland; 3 The Oncologic and Reconstructive Surgery Clinic, M.Sklodowska-Curie Institute–Oncology Center, Gliwice Branch, Gliwice, Poland

**Keywords:** Differentiated thyroid cancer, Radioiodine treatment, Low-risk differentiated thyroid cancer, Recurrence, Relapse

## Abstract

**Purpose:**

Although postoperative radioiodine (RAI) therapy has been used in patients with differentiated thyroid carcinoma (DTC) for many years, there is still lack of data defining the timing of RAI administration. A retrospective analysis was carried out to answer the question whether the time of postoperative RAI treatment demonstrated any impact on long-term outcomes, particularly in low-risk DTC.

**Material:**

The analyzed group involved 701 DTC patients staged pT_1b_-T_4_N_0_-N_1_M_0_, who underwent total thyroidectomy and postoperative RAI therapy. According to the time interval between DTC diagnosis and RAI administration, patients were allocated to one of three groups: up to 9 months (*N* = 150), between 9 and 24 months (*N* = 323), and > 24 months (*N* = 228). Median follow-up was 12.1 years (1.5–15.2).

**Results:**

Based on an initial DTC advancement and postoperative stimulated thyroglobulin concentration patients were stratified as a low-, intermediate-, and high-risk group. Low-risk patients, who received RAI therapy up to 9 months, demonstrated significantly lower risk of relapse comparing to those, in whom RAI was administered between 9 and 24 months and after 24 months since DTC diagnosis: 0%, 5.5%, and 7.1%, respectively. Regarding intermediate- and high-risk groups, the differences in the timing of postoperative RAI treatment were not significant.

**Conclusion:**

If postoperative RAI treatment is considered in low-risk DTC, any delay in RAI administration above 9 months since diagnosis may be related to poorer long-term outcomes.

## Introduction

Differentiated thyroid carcinoma (DTC) is the most common endocrine malignancy, characterized by a very good prognosis with 5-year overall survival rate of 98.5% [1]. A relatively indolent nature of the disease and the excellent outcomes raise a vibrant discussion, regarding the adequacy of treatment applied. The question, “whether DTC patients are overtreated or not”, which particularly refers to low-risk DTC, is reflected in the current ATA guidelines [2]. Comparing to previous one, the newest ATA guidelines are meaningfully less restrictive. In 2009, ATA recommended total or near-total thyroidectomy in all cases of thyroid cancer > 1 cm in diameter unless there were no contraindications for such procedure. Lobectomy alone was considered as sufficient treatment only in patients with small (< 1 cm), unifocal, intrathyroidal papillary thyroid carcinoma in the absence of prior head and neck irradiation or radiologically or clinically evident lymph node involvement [3]. In 2015, total thyroidectomy is definitely recommended only for patients with thyroid cancer > 4 cm, or if gross extrathyroidal extension, clinically apparent lymph node involvement or distant metastases are present. While in patients with lower local advancement including thyroid cancer > 1 cm and < 4 cm, clinically N0, without extrathyroidal extension, the initial surgical procedure can be either bilateral (total or near-total thyroidectomy) or unilateral one (lobectomy) [2]. Both above-mentioned 2015 ATA surgical recommendations are strong and supported by moderate quality of evidences. Similar changes concern the indications for postoperative radioiodine (RAI) administration. In 2009, postoperative RAI treatment was recommended in all patients with known distant metastases, gross extrathyroidal extension regardless of tumor size, primary tumor size > 4 cm in the absence of other risk factors and for selected patients with 1–4 cm intrathyroidal tumor and lymph node metastases, high-risk features or in those who demonstrated intermediate- or high-risk recurrence or DTC-related death. Such treatment was definitely not recommended for patients with unifocal or multifocal cancer < 1 cm if no high-risk features were present [3]. In 2015, postoperative RAI therapy should be routinely administered only in high-risk DTC; it is not recommended in low-risk DTC and should only be considered in ATA intermediate-risk group. Comparing 2009 and 2015 ATA guidelines, one should remember that ATA, in its current guidelines, redefined postoperative DTC risk group and widened low-risk category, including among others lymph node micrometastases or intrathyroidal follicular thyroid cancer with capsular and minimal vascular invasion [2]. In contrary to surgical guidelines, described above, the strength of current ATA recommendations regarding postoperative RAI treatment in low- and intermediate-risk DTC, except for unifocal papillary microcarcinoma, is weak and quality of evidences is low as studies evaluating the impact of postoperative RAI therapy, particularly in low-risk DTC, are retrospective or observational ones with a limited statistical power and their results are discrepant. Some papers reported a beneficial effect of postoperative RAI treatment on the risk of cancer recurrence or overall survival in low-risk DTC [4–7], whereas other studies not [8–12]. The results of systematic reviews did not demonstrate a significant impact of postoperative RAI therapy on the risk of DTC-related death, with conflicting data regarding DTC recurrence [13–15]. Interestingly, only recently apart from still opened question concerning postoperative RAI therapy “whether and whom to treat” another clinical problem has been raised in few studies: “when to treat” [16–19]? Indeed, there is lack of scientific data, which precisely define a timeframe between DTC diagnosis and postoperative RAI treatment. Therefore, we decided to carry out a retrospective analysis to answer the question whether the time of postoperative RAI administration had any effect on long-term outcomes in DTC, in particular in low-risk patients. We hypothesized that, if postoperative RAI therapy was not necessary in a low-risk group, any delay in RAI administration would not result in poorer outcomes in comparison to patients treated “on time”.

## Material and Methods

### Patients

The study group involved 701 DTC patients, staged pT_1b_-T_4_N_0_-N_1_M_0_, admitted for the first time due to thyroid carcinoma between 1994 and 1997, subjected to a retrospective analysis. All patients underwent total thyroidectomy within 1 year and postoperative RAI treatment within 2 years following DTC diagnosis. The group included 579 women (82.6%) and 122 (17.4%) men at a median age at DTC diagnosis of 44.8 years (range 6.9–77 years). Papillary thyroid cancer was diagnosed in 494 (70.5%) patients, whereas in the remaining 207 (29.5%) cases—follicular thyroid cancer. Two hundred and ninety-five patients (42.1%) demonstrated multifocal tumor growth, 90 (12.8%) patients thyroid capsule infiltration, and 99 (14.1%) vascular invasion. The median tumor diameter was 20 mm (range 1–150 mm). Based on the TNM classification (revised in 1997), T1 feature was present in 81 (11.5%) patients, T2 in 240 (34.2%) cases, whereas T3 and T4 in 43 (9.6%) and 48 (9.3%) patients, respectively. In the remaining 248 (35.4%) cases, Tx was stated. One hundred and fifty-four (22.0%) patients had lymph node metastases (N1). Median follow-up was 12.1 years (range 1.5–15.2) (Table 1).

**Table 1:** Characteristics of the study group

Characteristic	Number of patients (%)
Demographic data	
Women	579 (82.6)
Men	122 (17.4)
Median age at diagnosis	44.8 years (6.9–77 years)
Histopathological examination	
Papillary thyroid cancer	494 (70.5)
Follicular thyroid cancer	207 (29.5)
T1^a^	81 (11.5)
T2	240 (34.2)
T3	67 (9.6)
T4	65 (9.3)
Tx	248 (35.4)
Median tumor diameter	20 mm (range 1–150 mm)
Multifocal tumor growth	295 (42.1)
Vascular invasion	99 (14.1)
Thyroid capsule infiltration	90 (12.8)
N0, Nx	547 (78.1)
N1	154 (22.0)
M0	701 (100.0)
Treatment	
Total thyroidectomy	701 (100.0)
RAI ablation	701 (100.0)
Median follow-up	12.1 years (range 1.5–15.2 years)

^a^According to AJCC/TNM classification 1997

The first postoperative assessment, postoperative RAI therapy, and follow-up schemes were the same as in our previous analysis, published in 2016 [20].

### The First Postoperative Evaluation

The first postoperative evaluation was based on histopathological examination, and on results of neck ultrasound (US), TSH-stimulated serum thyroglobulin (Tg) measurement, and whole body scan (WBS) with a diagnostic RAI activity of 2 mCi (74 MBq). Such diagnostics, aimed to assess the postoperative staging, was carried out after 4-week thyroxine (LT4) withdrawal. In selected cases, additional studies, such as bone scan, FNAB, CT etc., were done, if indicated.

### Postoperative RAI Treatment

Postoperative RAI therapy was proceeded by 4–6-week LT4 withdrawal to achieve a required TSH level ≥ 25 IU/l. The median-administered RAI activity was 60 mCi (2220 MBq), range 27.8–150 mCi (1029–5550 MBq). WBS, together with a spot view of neck and chest, was done 72 h after RAI administration.

### Follow-Up

Further DTC monitoring was based on the evaluation of serum TSH, Tg with Tg recovery, or Tg antibodies during thyroxine administration and neck US every 6 months following postoperative RAI therapy. The first evaluation of treatment outcomes was done 6–12 months after postoperative RAI treatment and involved neck US, TSH-stimulated Tg measurement, diagnostic WBS, chest X-ray, and other imaging procedures, if indicated. Complete remission was confirmed if no RAI uptake on diagnostic WBS was observed, stimulated serum Tg level was ≤ 10 ng/ml at the absence of Tg antibodies, and neck US and other imaging studies were normal. Persistent disease was diagnosed in patients with structural DTC (abnormal neck US or other imaging studies, pathological RAI uptake in WBS, and/or stimulated serum Tg level > 30 ng/ml). In patients who did not fulfill criteria for complete remission or structural DTC, doubtful remission (stimulated Tg between 10 and 30 ng/ml with negative WBS and normal results of other examinations) was stated.

To evaluate the dependence between the time of postoperative RAI treatment and recurrence rate, patients were allocated to one of three groups, according to the time interval between DTC diagnosis and RAI administration: up to 9 months (*N* = 150), between 9 and 24 months (*N* = 323), and > 24 months (*N* = 228).

### Statistical Analysis

Statistical analysis was based on the calculation of disease-free survival (DFS) and freedom-to-progression time (FFP). DFS was defined as a time from the confirmation of complete DTC remission after surgery to disease relapse, death, or last follow-up. In patients, in whom complete remission was not obtained, DFS was coded as zero, while FFP was a time from the first diagnostics after surgery or postoperative RAI treatment to disease relapse or progression.

Time-to-event data were analyzed using Kaplan-Meier method and compared by log-rank, Breslow, and Tarone-Ware tests; the highest *p* value was reported. Quantitative variables were calculated for the association with time-to-event by Cox regression. *P* values below *p* < 0.05 were considered as statistically significant. Multivariate Cox regression was used, with stepwise backward feature elimination. The statistical was carried out by the use of IBM SPSS Statistics ver. 22 (IBM Corp., Armonk, New York, USA) and JMP ver. 10.0 (SAS Corp, Cary, North Carolina, USA).

## Results

### Multivariate Analysis

The first part of the study, published in 2016 [20], defined independent clinical factors influencing the risk of DTC recurrence by a multivariate Cox regression analysis for FFP. TSH-stimulated serum thyroglobulin (Tg), measured after total thyroidectomy before postoperative RAI treatment, was the most potent risk factor. If Tg values exceeded 30 ng/ml, the risk DTC relapse increased nearly six-fold (*p* = 0.000). Tg level between 10 and 30 ng/ml increased the risk of recurrence nearly 3 times (*p* = 0.017), whereas lower stimulated Tg levels, below 1 ng/ml and 1–10 ng/ml, did not show any negative impact on the risk of recurrence. Lymph node metastases at DTC diagnosis (N1) were related to a nearly 4-fold higher recurrence risk than in patients without lymph node involvement (N0 and Nx; *p* = 0.000). Other independent negative risk factors involved a larger tumor size > 4 cm (T3), extrathyroidal extension (T4) age above 60 years at DTC diagnosis, and low percentage of RAI uptake (T24) in thyroid bed before postoperative RAI treatment [20].

### Postoperative Risk Stratification of the Study Group

**Fig. 1:**
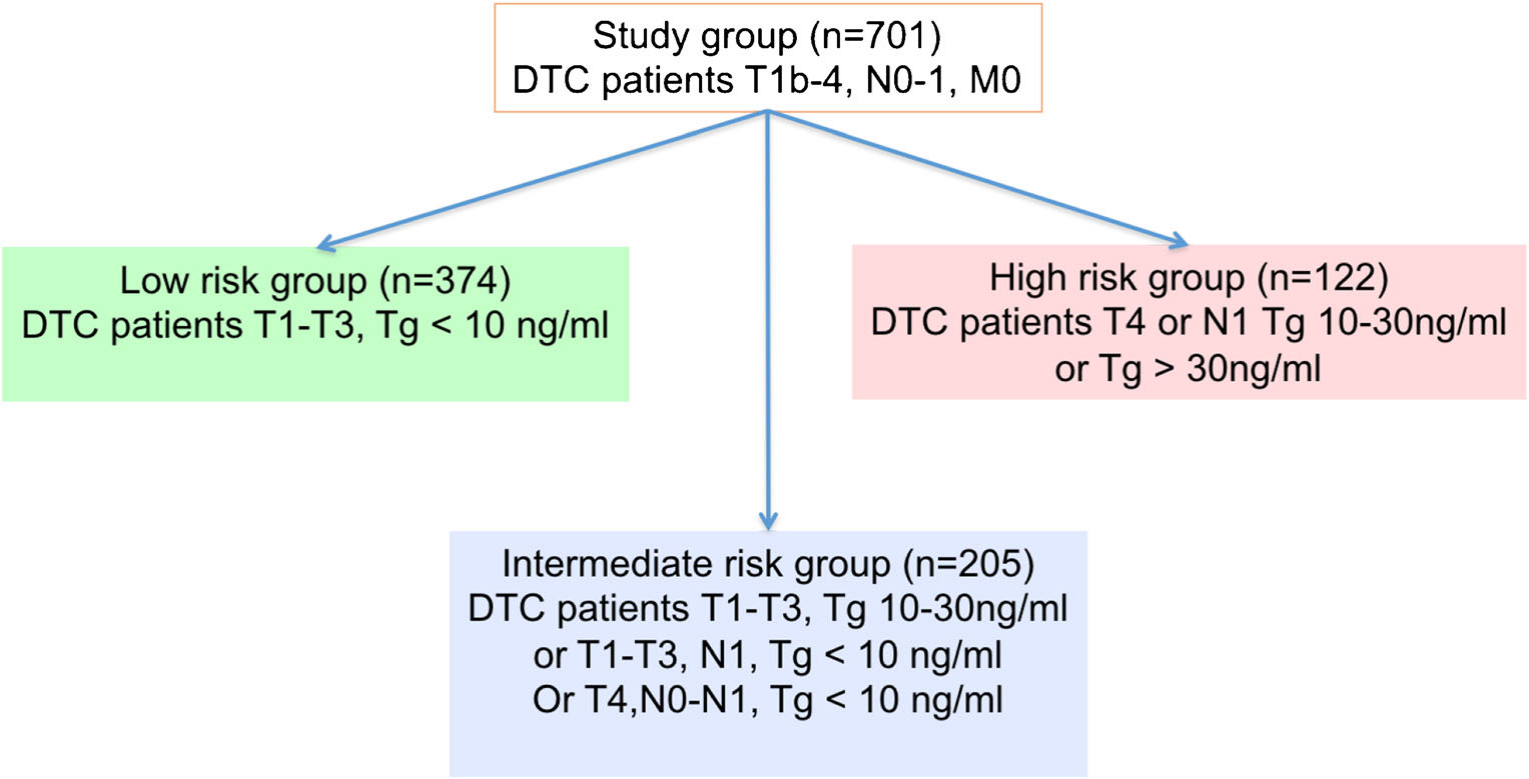
Postoperative risk stratification of the study group based on TNM staging and stimulated serum Tg concentration before complementary RAI treatment

**Fig. 2:**
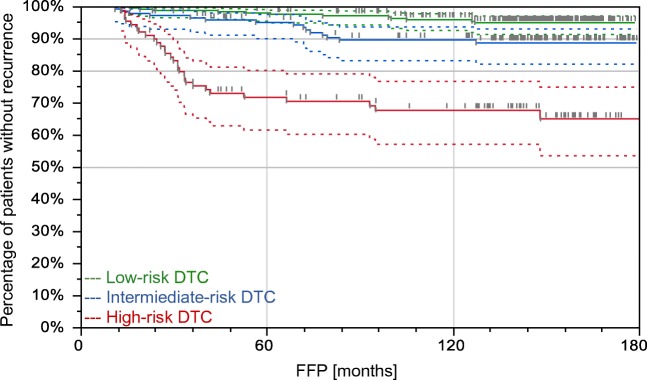
Differences in FFP (freedom from progression) were significant between the risk groups. The high-risk patients were characterized by the poorest prognosis

Before postoperative RAI treatment, the patients were stratified according to the factors selected in a multivariate Cox regression as low, intermediate, or high risk. Low-risk group involved 374 patients staged T1-T3N0Nx with stimulated Tg level < 10 ng/ml. Intermediate-risk group comprised of 205 patients staged either T1-T3 with stimulated Tg level 10–30 ng/ml or T1-T3N1 and T4N0N1 with stimulated Tg < 10 ng/ml. High-risk group included 122 patients with either stimulated Tg above 30 ng/ml or staged T4 or N1 with Tg level ranged between 10 and 30 ng/ml (Fig. 1). These risk groups showed significant differences in FFP (Fig. 2).

### The Impact of the Timing of Postoperative RAI Therapy on DTC Recurrence Risk

To answer the question whether the time of RAI administration significantly influences long-term outcomes, a group of patients treated with RAI up to 9 months from DTC diagnosis was compared to a group of patients in whom postoperative RAI therapy was carried out between 9 and 24 months or 24 months after the diagnosis.

**Fig. 3:**
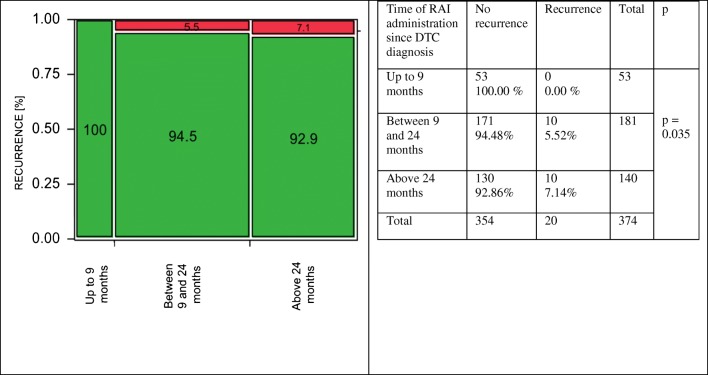
Low-risk DTC. Earlier RAI administration was related to significantly better long-term outcomes comparing to patients in whom the treatment was delayed. No DTC recurrence was observed in a subgroup of patients treated with RAI up to 9 months from DTC diagnosis, whereas when the RAI treatment was delayed, the risk of relapse was significantly higher 5.5% and 7.1% in patients treated between 9 and 24 months and above 24 months from DTC diagnosis, respectively (*p* = 0.035)

**Fig. 4:**
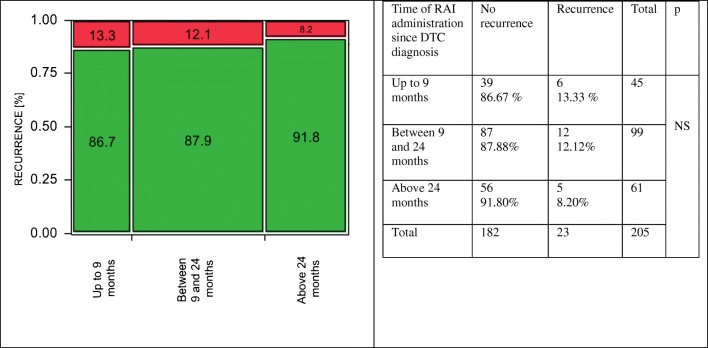
Intermediate-risk DTC. There were no significant differences regarding the risk of cancer recurrence depending on the time of RAI administration

**Fig. 5:**
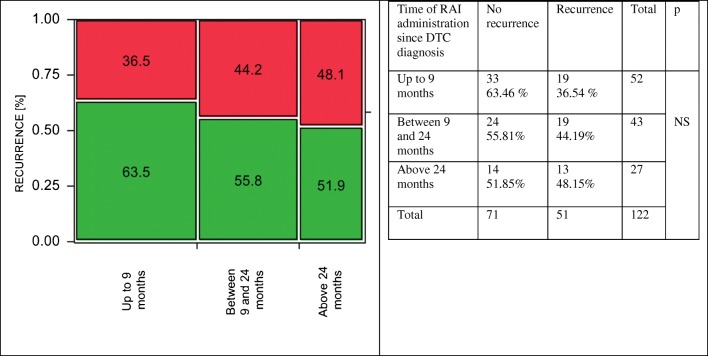
High-risk DTC. A beneficial trend towards an earlier RAI administration was noticed, but the differences between different time intervals of RAI administration were not significant

In low-risk DTC, the differences between the groups were significant (Cohran-Armitage test for trend, *p* = 0.035). The risk of recurrence was 0% (no events) in patients who received RAI therapy up to 9 months from DTC diagnosis, whereas in groups with delayed RAI administration, between 9 and 24 months and after 24 months: 5.5% and 7.1%, respectively (Fig. 3). In contrary to a low-risk group, the differences regarding the time of RAI administration were not significant both in intermediate- and high-risk DTC patients. In an intermediate-risk group, the lowest percentage of DTC recurrence was noticed in patients in whom RAI therapy was carried out above 24 months from the diagnosis (Fig. 4), whereas in a high-risk group, delayed RAI therapy was related to a slight but not statistically significant worsening of long-term outcomes. The recurrence rates were 36.5%, 44.2%, and 48.1% for patients treated up to 9 months, 9–24 months, and > 24 months from DTC diagnosis, respectively (Fig. 5). When the impact of treatment timing was assessed in the context of the risk class in the setting of a multivariate analysis, both factors were independently associated with DTC relapse, although the reliability of this analysis was limited by the lack of relapses in low-risk patients treated within 9 months (as the model was unstable, the data were not presented in the detail).

## Discussion

The data reporting the effect of RAI timing on long-term outcomes, published so far, are scarce. The present study, including 701 DTC patients who underwent total thyroidectomy and postoperative RAI treatment, demonstrated a significant impact of the time of RAI administration on recurrence rate in a low-risk group. If postoperative RAI therapy was carried out up to 9 months from DTC diagnosis, the risk of relapse decreased up to 0%. To our best knowledge, this is the first report demonstrating a positive dependence between the timing of postoperative RAI treatment and the recurrence risk, in particular in low-risk DTC patients. The analysis of the impact of the timing of postoperative RAI therapy on DTC final outcomes was possible thanks to a difficult situation we had to face up in Poland in the late nineties of XX century. That time, the patients had to wait in a long queue for postoperative RAI therapy and the vast majority of them were treated later than 6 months after surgery. Unfortunately, due to a very low number of deaths, we were not able to analyze the relationship between the time of RAI administration and overall survival.

In 2016, Suman et al. analyzed the impact of the timing of postoperative RAI therapy on overall survival in low- and intermediate-risk DTC [17]. The study involved a large number of DTC patients: 7.306 in low-risk group and 16.609 in intermediate-risk group. Postoperative RAI therapy was considered as early if it was carried out up to 3 months after thyroidectomy and as a delayed one if the treatment took place 3–12 months after surgery. Overall survival at 5 years and 10 years did not differ significantly between the “early” and “delayed” groups in both low- and intermediate-risk DTC [17]. Another paper, published by Greek authors in 2014, did not report any significant differences regarding the time of postoperative RAI therapy and long-term outcomes in low-risk DTC either. The authors analyzed a group of 107 low-risk patients. Nearly 47% of patients received RAI activity in less than 4.7 months (median 3.0; range 0.8–4.7 months), whereas 53% in more than 4.7 months (median 6.0; range 4.8–30.3 months) after near-total thyroidectomy. At the median follow-up of 87.3 months (range 23.3–251.6 months), all patients were disease free, regardless of the time of RAI administration [16]. Similar observation was reported by a Brazilian group in 2016 [19]. This study involved 901 DTC patients, among them 228 patients classified as ATA low risk. The median interval between total thyroidectomy and postoperative RAI treatment was 6 months (range 3–10 months). A group of patients with postoperative RAI therapy ≤ 6 months (group A) was compared to a group treated > 6 months following surgery (group B). The median time interval in group A was 3 months (range 2–5 months), whereas in group B, 10.5 months (range 8–16 months). One year after initial therapy, 59.3% of patients from a group A and 65.6% of patients from a group B were disease free. This difference was not significant. These findings did not significantly change after a longer median follow-up of 6 years: 63.3% of patients vs. 67.7%, respectively. In addition, there was no difference in recurrence rate between the groups (5.4% in group A and 3% in group B). The percentage of low-risk patients was 36.6% in group A and 48.0% in group B. Unfortunately, the authors did not analyze low-risk patients separately [19].

Regarding intermediate- and high-risk groups, our findings are in concordance with published data. We failed to demonstrate any beneficial effect of earlier postoperative RAI therapy on DTC long-term outcomes. Surprisingly, among intermediate-risk patients, the lowest percentage of DTC recurrences was noticed in a group treated with RAI > 24 months after DTC diagnosis. Such findings probably reflect a proper qualification process for postoperative RAI therapy. At that time in our center, patients with less advanced DTC stages waited longer for the treatment. Suman et al., in a paper mentioned above, did not find any differences in 10-year overall survival between early and delayed RAI administration: 95.3% and 95.9%, respectively.

Considering high-risk patients, we observed only a beneficial trend towards an earlier RAI administration and the recurrence rate, but the differences between distinct time intervals were not significant. According to another report, published by the American authors, there was no significant difference in overall survival between earlier and delayed RAI treatment in high-risk patients either [18].

We are aware that a high percentage of Tx patients (35.4%) is an important limitation of our study. Most patients from the study group underwent primary thyroid surgery between 1994 and 1997, some of them even earlier. The majority of them had two-stage total thyroidectomy. Moreover, in such cases, the primary surgery was carried out usually outside our center. Simultaneously, histopathological report that time was less accurate than current assessment. Therefore, if any doubts existed, Tx feature was diagnosed. Nevertheless, we would like to add that the differences in recurrence rate in Tx group regarding the time of RAI administration are similar to a low-risk class (data not shown).

Another important issue is related to the risk stratification, used in our study. It is based on the results of a multivariate analysis, given in our previous paper [20]. It involved DTC stage (T and N features) and a postoperative stimulated thyroglobulin level. This stratification reflects our management, used in the time, when the patients were treated (between 1994 and 1997). We believe that ATA classification would be strongly biased by the absence of precise tumor diameter data, mandatory for such stratification.

Moreover, the papers discussed above used different time point to evaluate the role of postoperative RAI therapy. In our analysis, the use of shorter cut-off points at 3 or 6 months after DTC diagnosis was not possible due to too low number of patients in subgroups to reach a statistical significance. Relatively high number of patients were treated latter than 2 years after DTC diagnosis, what was related usually to low DTC advancement or sometimes patient preference. The first evaluation of treatment outcomes was carried out 6–12 months after postoperative RAI therapy. However, the patients were followed up much longer. The analysis summarizes the whole follow-up period, not only up to 6–12 months after postoperative RAI therapy.

## Conclusions

Our study was not aimed to be a voice in the ongoing discussion, whether postoperative RAI therapy is necessary in low-risk DTC or not. This is the role of currently ongoing prospective randomized clinical trials. But it is worthy to emphasize that, if postoperative RAI treatment is considered in low-risk DTC patients, any delay in RAI administration above 9 months from cancer diagnosis may be related to poorer long-term outcomes.
